# Return Cases of Scarlet Fever

**Published:** 1898-09-24

**Authors:** 


### RETURN CASES OF SCARLET FEVER.

Commenting upon an article by Dr. Killick Millard, to which we recently drew attention, Dr. Clement Dukes, of Rugby, suggests a mode in which the infection of scarlet fever may be carried by convalescents, which may, perhaps, explain the comparative frequency with which "return cases" were traced by Dr. Millard to convalescents who still had a certain amount of rhinitis at the time of their discharge. The theory which Dr. Dukes holds is that that even after a patient has ceased to be himself infectious, *i.e.*, has lost all the infectiousness which has arisen from his own disease, he may still, if placed in the same room with a recent case, become a carrier of infection, his nasal cavities and fauces becoming "saturated with the fresh virus of scarlet fever." This is quite on all fours with what we know to happen to the bacillus of diphtheria, and the point is well worth remembering and testing by the light of experience. According to Dr. Dukes, convalescents must never mix with recent cases, or they may become the carriers of infection, although they themselves may be quite well.

